# Impact of Paravalvular Leak on Outcomes After Transcatheter Aortic Valve Implantation: Meta-Analysis of Kaplan-Meier-derived Individual Patient Data

**DOI:** 10.1016/j.shj.2022.100118

**Published:** 2022-11-14

**Authors:** Michel Pompeu Sá, Xander Jacquemyn, Jef Van den Eynde, Panagiotis Tasoudis, Ozgun Erten, Serge Sicouri, Francisco Yuri Macedo, Tilak Pasala, Ryan Kaple, Alexander Weymann, Arjang Ruhparwar, Marie-Annick Clavel, Philippe Pibarot, Basel Ramlawi

**Affiliations:** a Department of Cardiothoracic Surgery, Lankenau Heart Institute, Lankenau Medical Center, Main Line Health, Wynnewood, Pennsylvania, USA; b Department of Cardiothoracic Surgery Research, Lankenau Institute for Medical Research, Wynnewood, Pennsylvania, USA; c Department of Cardiovascular Sciences, KU Leuven, Leuven, Belgium; d Division of Structural Heart Disease, Department of Medicine, Hackensack University Medical Center, Hackensack, New Jersey, USA; e Department of Thoracic and Cardiovascular Surgery, West German Heart and Vascular Center Essen, University Hospital of Essen, University of Duisburg-Essen, Essen, Germany; f Quebec Heart & Lung Institute, Université Laval, Québec City, Québec, Canada; g Department of Medicine, Faculty of Medicine, Université Laval, Québec City, Québec, Canada

**Keywords:** Aortic valve disease, Cardiac surgical procedures, Cardiovascular surgical procedures, Heart valve prosthesis implantation, Meta-analysis, Transcatheter aortic valve replacement, heart valves

## Abstract

**Background:**

Paravalvular leak (PVL) after transcatheter aortic valve implantation (TAVI) is frequent and the impact of mild PVL on outcomes remains uncertain. Our study aimed to evaluate the impact of PVL on TAVI outcomes.

**Methods:**

To analyze late outcomes of patients after TAVI according to the presence and severity of PVL, PubMed/MEDLINE, EMBASE and Google Scholar were searched for studies that reported rates of all-cause mortality/survival and/or rehospitalization and/or cardiovascular mortality accompanied by at least one Kaplan-Meier curve for any of these outcomes. We adopted a 2-stage approach to reconstruct individual patient data based on the published Kaplan-Meier graphs.

**Results:**

Thirty-eight studies with Kaplan-Meier curves met our eligibility criteria including over 25,000 patients. Patients with any degree of PVL after TAVI had a significantly higher risk of overall mortality (hazard ratio (HR), 1.52; 95% confidence interval (CI), 1.43-1.61; *p* < 0.001), rehospitalization (HR, 1.81; 95% CI, 1.54-2.12; *p* < 0.001), and cardiovascular mortality (HR, 1.52; 95% CI, 1.33-1.75; *p* < 0.001) over time. These findings remained consistent when we stratified the results for the methods of assessment of PVL (i.e., echocardiography vs. angiography) and PVL severity. Both moderate/severe PVL and mild PVL were associated with increased risk of overall mortality (*p* < 0.001), rehospitalization (*p* < 0.001), and cardiovascular mortality (*p* < 0.001) during follow-up.

**Conclusions:**

Patients with PVL, even if mild, experience higher risk of all-cause mortality, rehospitalization, and cardiovascular mortality following TAVI. These findings provide support to the implementation of procedural strategies to prevent any degree of PVL at the time of TAVI.

## Graphical abstract

**Figure:**
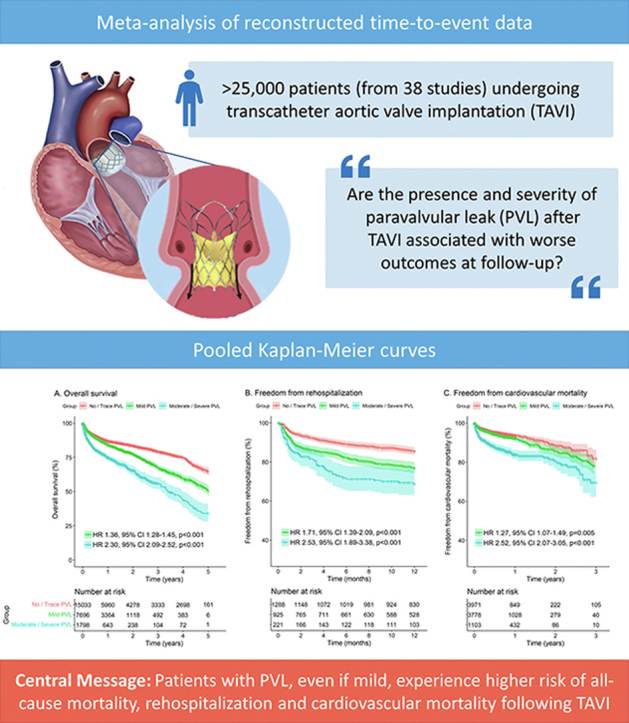

## Introduction

Paravalvular leak (PVL) after transcatheter aortic valve implantation (TAVI) is common and is reported to occur more frequently than after surgical aortic valve replacement (SAVR).^1^ Moderate/severe PVL has been associated with 2- to 3-fold increase in the risk of all-cause mortality.^2^ With the development of newer generation transcatheter heart valves (THVs), the incidence of moderate/severe PVL has declined substantially and has become close to that of SAVR.^3^ However, the incidence of moderate/severe PVL remains higher in TAVI vs. SAVR in some subset of patients such as those with bicuspid aortic valve (as compared with patients with tricuspid aortic valve),^4^, ^5^, ^6^ those who receive self-expandable valves (in comparison with balloon-expandable valves)^7^ and those who have left ventricle outflow tract (LVOT) calcification.^8^ Furthermore, the rates of mild PVL remain much more frequent in TAVI vs. SAVR, even with the new-generation THVs.^9^ The impact of mild PVL on clinical outcomes after TAVI remains unclear and led to conflicting results in the literature.

The objective of this study was to evaluate the association between the presence and severity of PVL after TAVI and the risk of all-cause mortality, rehospitalization, and cardiovascular mortality in the follow-up. We performed a pooled analysis of Kaplan–Meier-estimated individual patient data (IPD) from studies comparing the outcomes of patients with and without PVL after TAVI.

## Methods

### Eligibility Criteria, Databases, and Search Strategy

This study followed the Preferred Reporting Items for Systematic Reviews and Meta-Analyses (PRISMA) reporting statement.^10^ Using the *P*opulation, *E*xposure, *C*omparison, *O*utcome, and *S*tudy design (PECOS) strategy, studies were included if the following criteria were fulfilled:

1) The population comprised adults who suffered from aortic valve disease treated with TAVI;
2) There was a group of patients exposed to any PVL after TAVI (mild, moderate, or severe);
3) There was a group of patients who had none/trace PVL;
4) The study included outcomes such as survival/mortality and/or rehospitalization and/or cardiovascular mortality with at least one of these outcomes with Kaplan-Meier curves;
5) The study design was retrospective/prospective, randomized/non-randomized, mono/multicentric, with matched/unmatched populations.

The following sources were searched for articles meeting our inclusion criteria and published by April 30, 2022: PubMed/MEDLINE, EMBASE, and Google Scholar, and the reference lists of relevant articles. We searched for the following terms: [“transcatheter aortic valve replacement” OR “transcatheter aortic valve implantation” OR “TAVI” OR “TAVR”] AND [“aortic valve insufficiency” OR “aortic valve incompetence” OR “aortic valve regurgitation” OR “paravalvular leakage” OR “paravalvular leak” OR “paravalvular regurgitation” OR “perivalvular leakage” OR “perivalvular leak” OR “perivalvular regurgitation”]. Studies were selected by 2 independent reviewers. When there was disagreement, a third reviewer made the decision to include or exclude the study.

### Assessment of Risk of Bias

The Risk of Bias in Non-Randomized Studies of Interventions tool (ROBINS-I) was systematically used to assess included studies for risk of bias.^11^ Two independent reviewers assessed risk for bias. When there was a disagreement, a third reviewer checked the data and made the final decision.

### Statistical Analysis

Most authors pool their data using mostly random-effects models to produce incidence rate ratios (IRRs), odds ratios (ORs), or risk ratios (RRs) as summary measures. Time-to-event outcomes are not easily incorporated into traditional meta-analyses. Researchers have resorted to pooling median survival times, IRR or event rates estimated from survival estimates at given timepoints or made direct estimates of the hazard ratios (HRs) across the studies. All these approaches have been shown to be limiting and unsatisfactory, as they do not allow the production of pooled Kaplan-Meier curves and fail to recognize some of the central tenets of survival analysis such as censoring and the proportional hazards assumption.^12^ In response to inconsistent reporting that resulted from these diverging approaches, the “curve approach” has emerged as the current standard for meta-analysis of aggregated time-to-event data.^13^ This approach reconstructs IPD based on the published Kaplan-Meier graphs from the included studies.

We used the two-stage approach as described by Liu et al^14^ based on the R package “IPDfromKM” (version 0.1.10). In the first stage, raw data coordinates (time, survival probability) were extracted from each treatment arm in each of the Kaplan-Meier curves. In the second stage, the data coordinates were processed based on the raw data coordinates from the first stage in conjunction with the numbers at risk at given timepoints, and IPD were reconstructed. Finally, the reconstructed IPD from all studies were merged to create the study dataset. The cumulative incidence of each outcome at follow-up in both arms (with and without PVL after TAVI) was visually assessed using Kaplan-Meier estimates with the R packages “survival” (version 3.2-13) and “survminer” (version 0.4.9). HRs with 95% confidence intervals (CIs) for the difference between both treatment arms were calculated using a Cox regression model with the R package “coxphw” (version 4.0.2). HR > 1 (with *p*-value < 0.05) would indicate a higher risk of mortality or rehospitalization with the presence of PVL. The proportionality of the hazards of each Cox model was checked with the Grambsch-Therneau test and diagnostic plots based on Schoenfeld residuals. All analyses were completed with R Statistical Software (version 4.1.1, Foundation for Statistical Computing, Vienna, Austria).

## Results

### Study Selection and Characteristics

**Figure 1:**
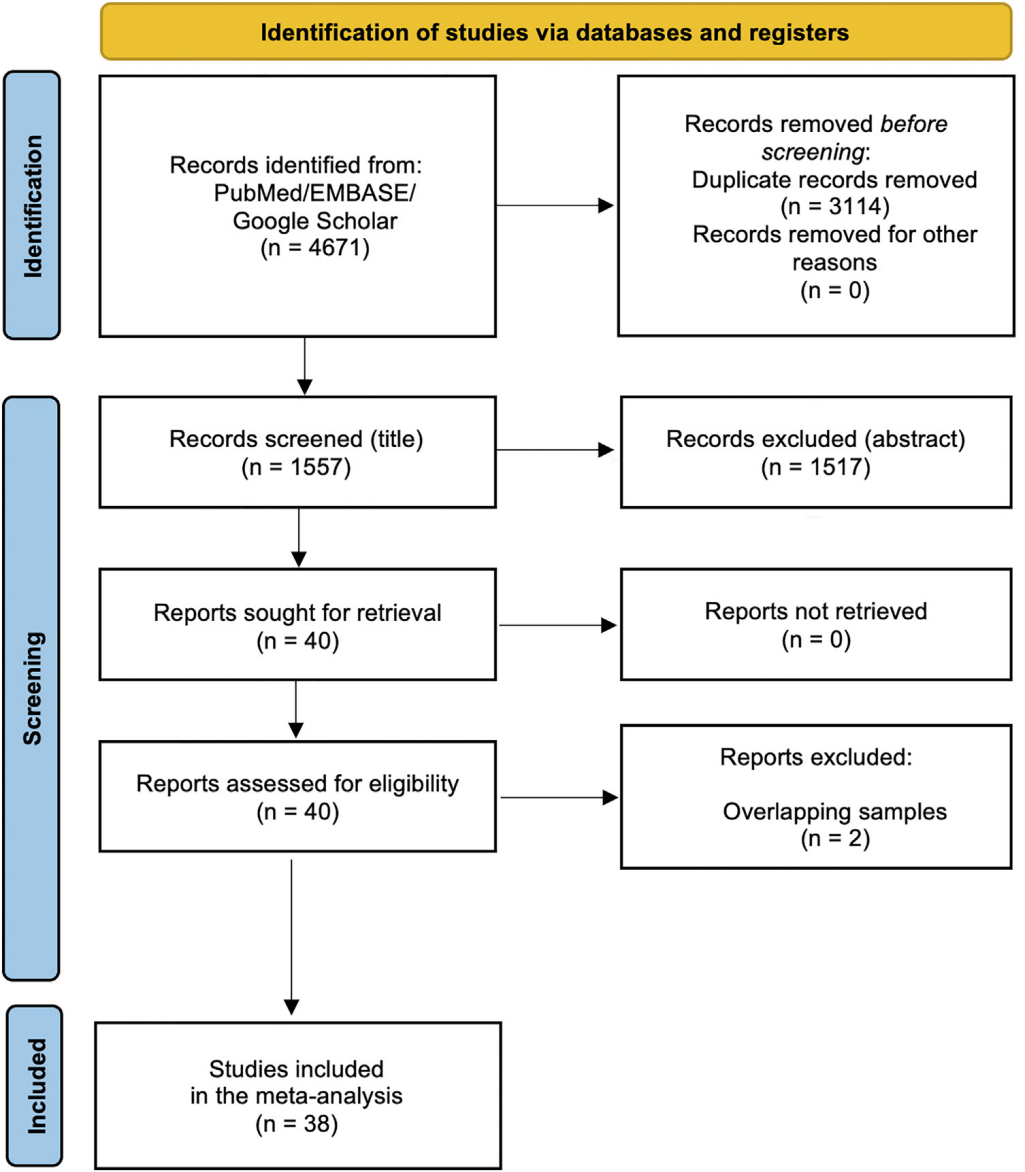
Flow diagram of studies included in data search.

**Figure 2:**
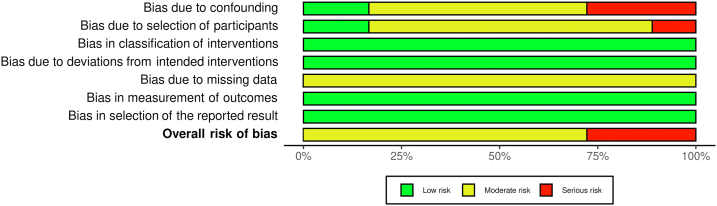
Risk of bias summary–ROBINS-I tool with summary plot. ROBINS-I, Risk of Bias in Non-Randomized Studies of Interventions.

After excluding duplicates and non-eligible studies, 38 studies [Supplemental Reference List] met our eligibility criteria (Figure 1). Almost all the studies were non-randomized, while 23 studies were multicentric and 9 studies included prospective populations (Supplemental Table 1). All the studies included patients with a mean age of around 80 years. Figure 2 shows the qualitative assessment of the studies with the summary plots generated by the ROBINS-I tool. We have some concerns related to missing data and confounding factors since most of studies comprised unadjusted cohorts, which are more prone to biases.

### Analysis of Outcomes According to the Presence of Any Grade of PVL (Measured by Echocardiography)

**Figure 3:**
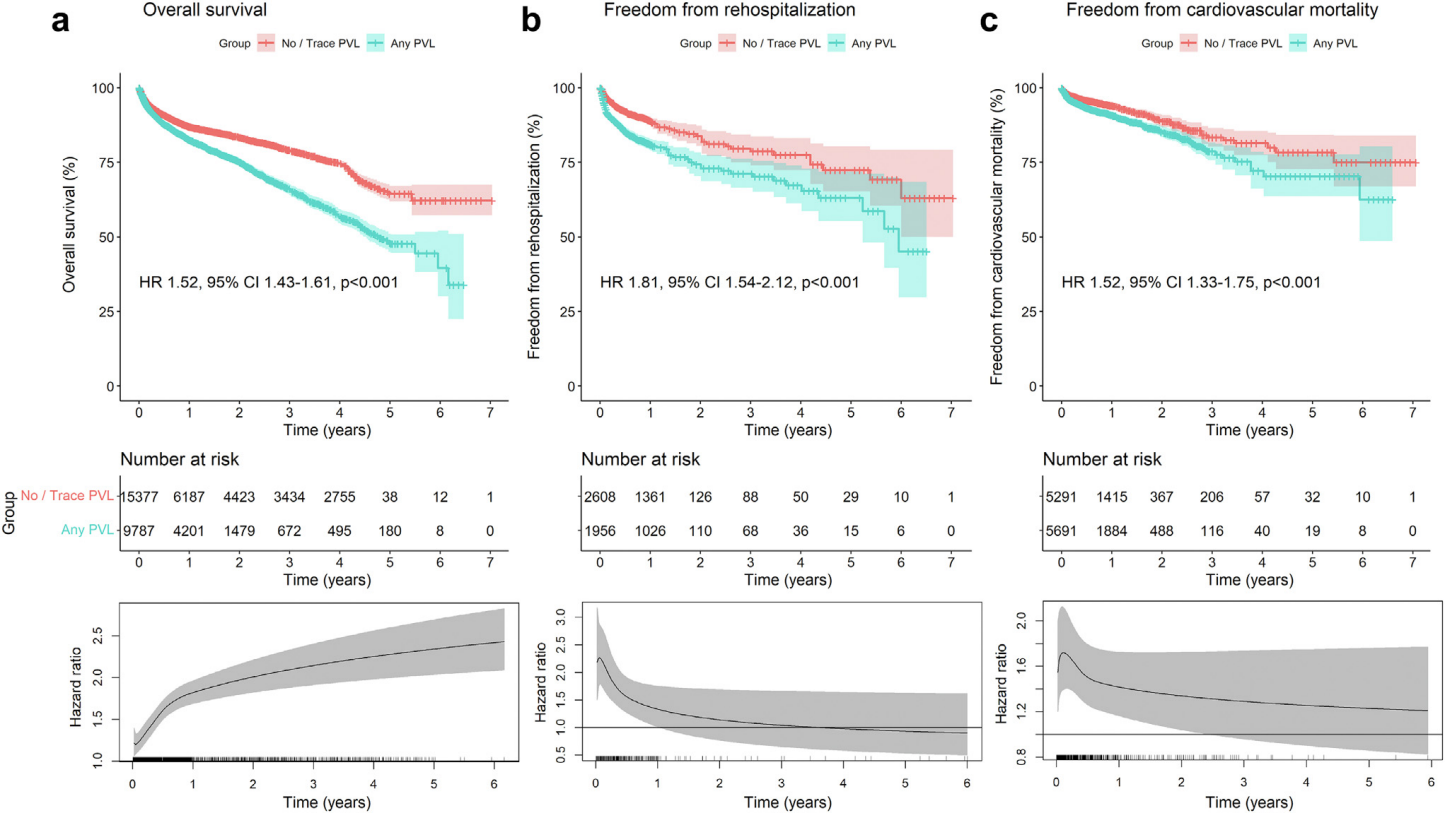
Pooled Kaplan-Meier curves showing the cumulative risk of all-cause mortality (a), rehospitalization (b), and cardiovascular death (c) for patients with any degree of PVL measured by echocardiography. Pooled Kaplan-Meier curves accompanied by their respective time-varying HRs with 95% CI at every given time during follow-up; these are derived from flexible parametric survival models with B-splines. Abbreviations: CI, confidence interval; HR, hazard ratio; PVL, paravalvular leak.

Figure 3 depicts the pooled Kaplan-Meier curves for the cumulative risk of overall mortality (Figure 3a), rehospitalization (Figure 3b), and cardiovascular mortality (Figure 3c) in all studies including data on these outcomes according to the presence of any PVL. Flexible parametric survival models were performed for all models to estimate the time-varying HRs with 95% CIs for every given timepoint during follow-up.

To conduct the analysis for cumulative risk of overall mortality (Figure 3a), the data of 25,164 patients (no/trace PVL: 15,377 patients; any PVL: 9787 patients) from 22 studies were pooled. Patients with any grade of PVL had a significantly higher risk of death (HR, 1.52; 95% CI, 1.43-1.61; *p* < 0.001). However, in this Cox model, the proportional hazards assumption was violated. Therefore, this model might be underestimating the actual impact of PVL on mortality. The flexible parametric model, which estimates time-varying HRs with 95% CIs for every given timepoint during follow-up, shows an increasing HR over time. This suggests that the presence of any grade of PVL has an increasing impact on risk of mortality over time.

Similarly, for the analysis for cumulative risk of rehospitalization (Figure 3b) the data of 4564 patients (no/trace PVL: 2608 patients; any PVL: 1956 patients) from 3 studies were pooled. Here, patients with any grade of PVL had a significantly higher risk of rehospitalization (HR, 1.81; 95% CI, 1.54-2.12; *p* < 0.001). In this Cox model as well, the proportional hazards assumption was violated. Therefore, this model might be underestimating the actual impact of PVL on rehospitalization. The flexible parametric model, which estimates time-varying HRs with 95% CIs for every given timepoint during follow-up, shows a peak of risk during the first months after TAVI, which stabilizes after 3 years toward HR = 1.

Lastly, for the analysis for cumulative risk of cardiovascular mortality (Figure 3c) the data of 10,982 patients (no/trace PVL: 5291 patients; any PVL: 5691 patients) from 7 studies were pooled. Patients with any grade of PVL had a significantly higher risk of cardiovascular mortality (HR, 1.52; 95% CI, 1.33-1.75; *p* < 0.001).

### Analysis of Outcomes According to the Presence Of Moderate/Severe PVL (Measured by Echocardiography)

**Figure 4:**
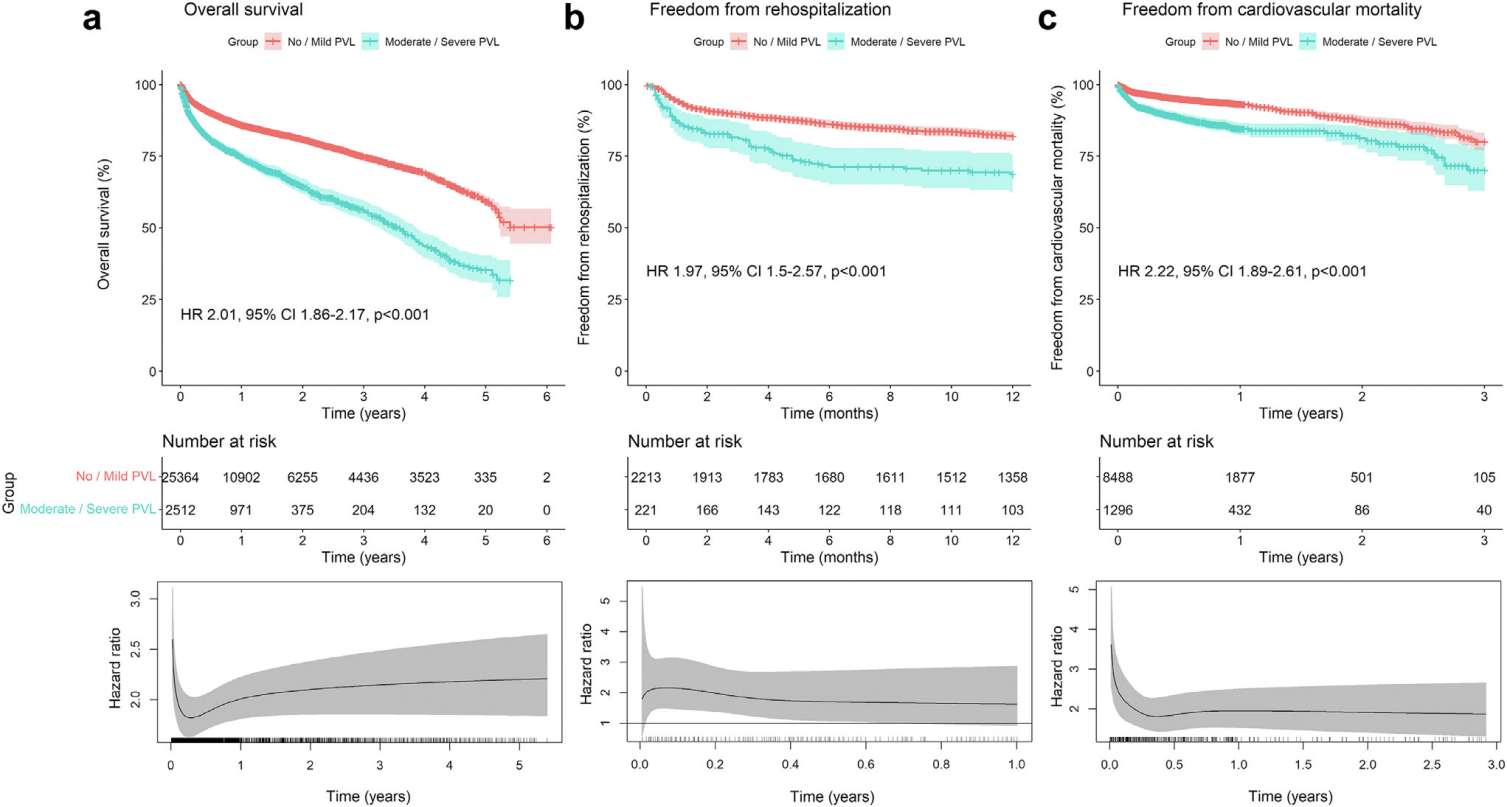
Pooled Kaplan-Meier curves showing the cumulative risk of all-cause mortality (a), rehospitalization (b), and cardiovascular death (c) for patients with moderate/severe PVL measured by echocardiography. Pooled Kaplan-Meier curves accompanied by their respective time-varying HRs with 95% CI at every given time during follow-up; these are derived from flexible parametric survival models with B-splines. Abbreviations: CI, confidence interval; HR, hazard ratio; PVL, paravalvular leak.

Figure 4 depicts the pooled Kaplan-Meier curve for the cumulative risk of overall mortality (Figure 4a), rehospitalization (Figure 4b), and cardiovascular mortality (Figure 4c) in all studies including data on these outcomes according to the presence of moderate/severe PVL vs. no/mild PVL. Flexible parametric survival models were performed for all models to estimate the time-varying HRs with 95% CIs for every given timepoint during follow-up.

For the analysis for cumulative risk of mortality (Figure 4a) the data of 27,876 patients (no/mild PVL: 25,364 patients; moderate/severe PVL: 2512 patients) from 30 studies were pooled. Patients with moderate/severe PVL had a significantly higher risk of mortality (HR, 2.01; 95% CI, 1.86-2.17; *p* < 0.001).

For the analysis for cumulative risk of rehospitalization (Figure 4b) the data of 2434 patients (no/mild PVL: 2213 patients; moderate/severe PVL: 221 patients) were pooled. Patients with moderate/severe PVL had a significantly higher risk of rehospitalization (HR, 1.97; 95% CI, 1.5-2.57; *p* < 0.001).

For the analysis for cumulative risk of cardiovascular mortality (Figure 4c) the data of 9784 patients (no/mild PVL: 8488 patients; moderate/severe PVL: 1296 patients) from 7 studies were pooled. Patients with moderate/severe PVL had a significantly higher risk of cardiovascular mortality compared to patients without or with mild/trace PVL (HR, 2.22; 95% CI, 1.89-2.61; *p* < 0.001).

### Analysis of Outcomes According to the Presence of Mild PVL (Measured by Echocardiography)

**Figure 5:**
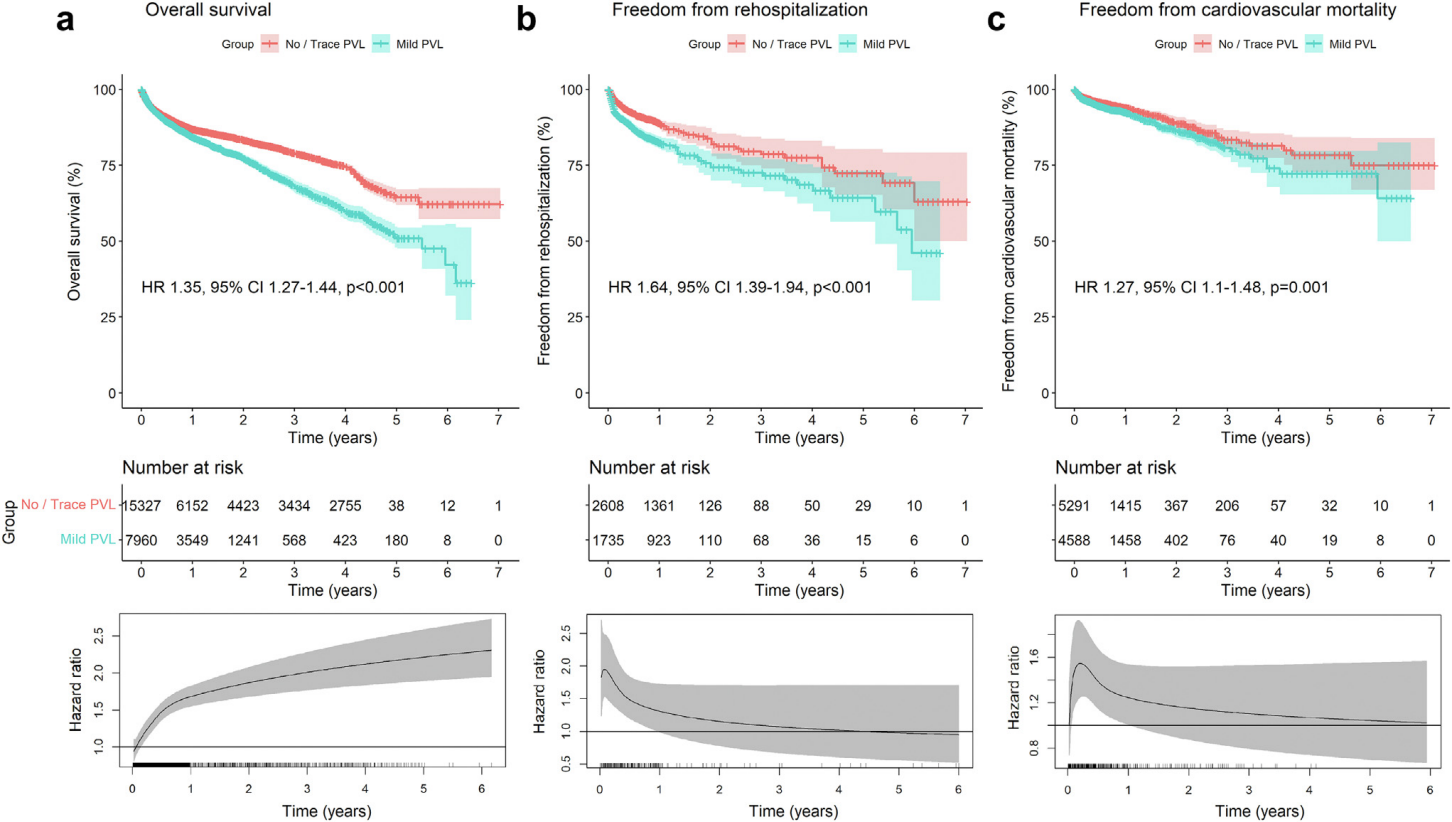
Pooled Kaplan-Meier curves showing the cumulative risk of all-cause mortality (a), rehospitalization (b), and cardiovascular death (c) for patients with mild PVL measured by echocardiography. Pooled Kaplan-Meier curves accompanied by their respective time-varying HRs with 95% CI at every given time during follow-up; these are derived from flexible parametric survival models with B-splines. Abbreviations: CI, confidence interval; HR, hazard ratio; PVL, paravalvular leak.

Figure 5 depicts the pooled Kaplan-Meier curve for the cumulative risk of overall mortality (Figure 5a), rehospitalization (Figure 5b), and cardiovascular mortality (Figure 5c) in all studies including data on these outcomes according to the presence of mild PVL vs. no/trace PVL. Flexible parametric survival models were performed for all models to estimate the time-varying HRs with 95% CIs for every given timepoint during follow-up.

For the analysis for cumulative risk of overall mortality (Figure 5a) the data of 23,287 patients (no/trace PVL: 15,327 patients; mild PVL: 7960 patients) from 21 studies were pooled. Patients with mild PVL had a significantly higher risk of mortality (HR, 1.35; 95% CI, 1.27-1.44; *p* < 0.001). Of note, in this Cox model, the proportional hazards assumption was also violated. Therefore, this model might be underestimating the actual impact of mild PVL on mortality. The flexible parametric survival model, which estimates time-varying HRs with 95% CIs for every given timepoint during follow-up, shows an increasing HR over time. This suggests that the presence of mild PVL has an increasing impact on risk of mortality over time.

For the analysis for cumulative risk of rehospitalization (Figure 5b) the data of 4564 patients (no/trace PVL: 2608 patients; mild PVL: 1735 patients) from 3 studies were pooled. Patients with mild PVL had a significantly higher risk of rehospitalization (HR, 1.64; 95% CI, 1.39-1.94; *p* < 0.001).

For the analysis for cumulative risk of cardiovascular mortality (Figure 5c) the data of 10,982 patients (no/trace PVL: 5291 patients; mild PVL: 4588 patients) from 7 studies were pooled. Patients with mild PVL had a significantly higher risk of cardiovascular mortality (HR, 1.27; 95% CI, 1.1-1.48; *p* = 0.001).

### Analysis of Outcomes According to the Presence of PVL (Measured by Echocardiography) With a 3-arm Comparison

**Figure 6:**
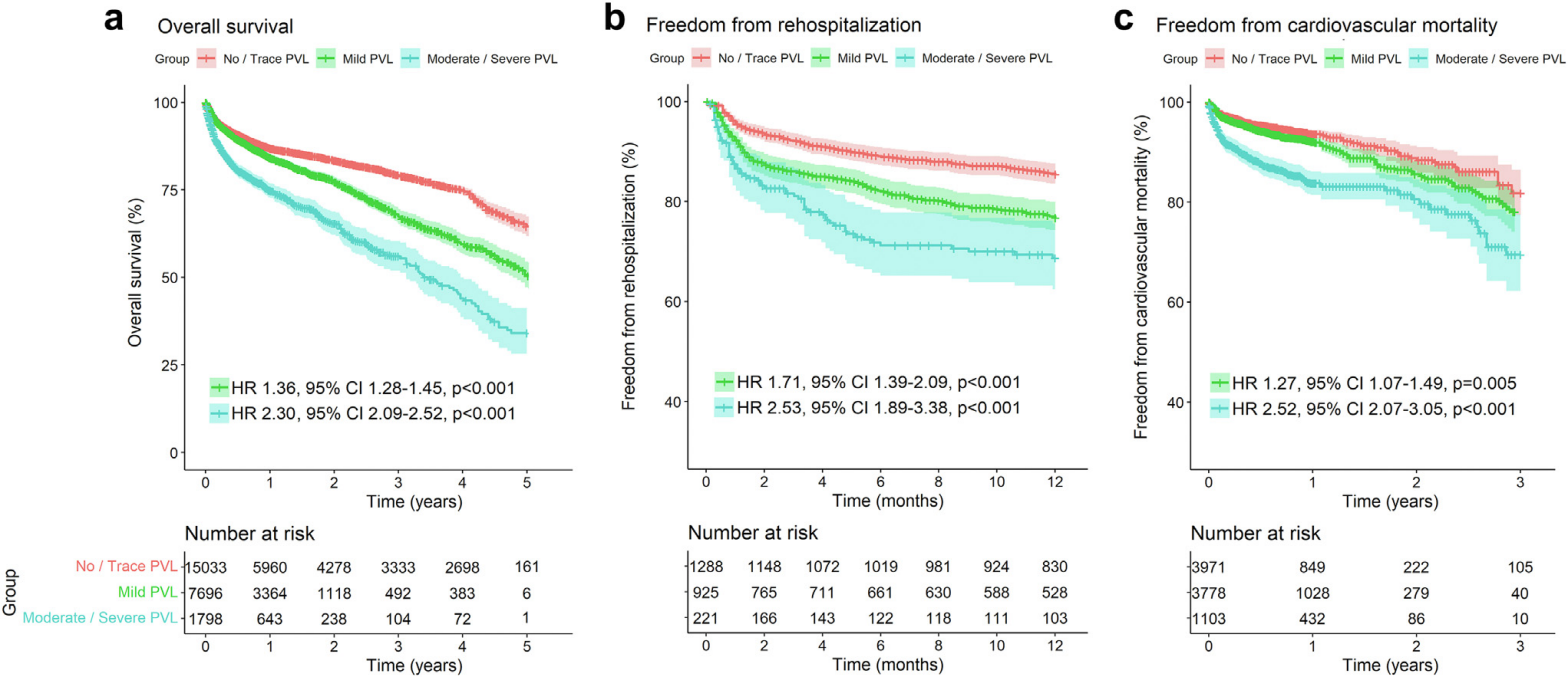
Pooled Kaplan-Meier curves showing the cumulative risk of all-cause mortality (a), rehospitalization (b), and cardiovascular death (c) with a 3-arm comparison according to PVL degrees measured by echocardiography. Abbreviations: CI, confidence interval; HR, hazard ratio; PVL, paravalvular leak.

Figure 6 depicts the pooled Kaplan-Meier curves for the cumulative risk of mortality, rehospitalization, and cardiovascular mortality in all studies including data on these outcomes according to the presence of PVL.

To conduct the analysis for the cumulative risk of mortality (Figure 6a), the data of 24,527 patients (no/trace PVL: 15,033 patients; mild PVL: 7696 patients; moderate/severe PVL: 1798) from 19 studies were pooled. Patients with any degree of PVL had a significantly higher risk of mortality compared to patients with no/trace PVL. This risk increases with increasing grade of paravalvular leakage (no/trace PVL vs mild PVL; HR, 1.36; 95% CI, 1.28-1.45; *p* < 0.001 and no/trace PVL vs moderate/severe PVL; HR, 2.30; 95% CI, 2.09-2.52; *p* < 0.001).

Similarly, in the analysis for cumulative risk of rehospitalization (Figure 6b) the data of 2434 patients (no/trace PVL: 1288 patients; mild PVL: 925 patients; moderate/severe PVL: 221) were analyzed. Here, patients with any degree of PVL had a significantly higher risk of rehospitalization compared to patients with no/trace PVL. This risk increases with increasing degree of PVL (no/trace PVL vs mild PVL; HR, 1.71; 95% CI, 1.39-2.09; *p* < 0.001 and no/trace PVL vs moderate/severe PVL; HR, 2.53; 95% CI, 1.89-3.38; *p* < 0.001).

Lastly, for the analysis for cumulative risk of cardiovascular mortality (Figure 6c) the data of 8852 patients (no/trace PVL: 3971 patients; mild PVL: 3778 patients; moderate/severe PVL: 1103) from 5 studies were pooled. Patients with any degree of PVL had a significantly higher risk of cardiovascular mortality compared to patients with no/trace PVL. This risk increases with increasing degree of PVL (no/trace PVL vs mild PVL; HR, 1.27; 95% CI, 1.07-1.49; *p* = 0.005; no/trace PVL vs moderate/severe PVL; HR, 2.52; 95% CI, 2.07-3.05; *p* < 0.001).

### Analysis of Outcomes According to the Presence of PVL Measured by Angiography

**Figure 7:**
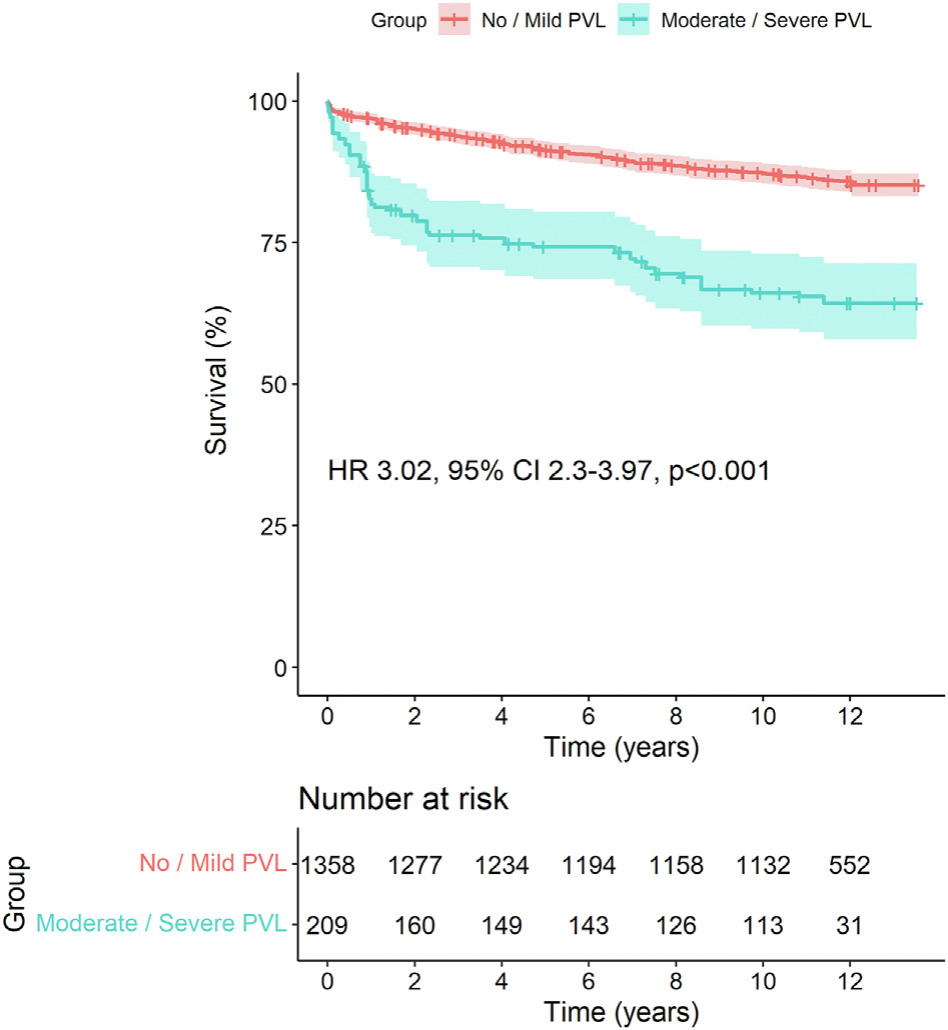
Pooled Kaplan-Meier curves showing the cumulative risk of all-cause mortality for patients with moderate/severe PVL measured by angiography. Abbreviations: CI, confidence interval; HR, hazard ratio; PVL, paravalvular leak.

Figure 7 depicts the pooled Kaplan-Meier curve for the cumulative risk of overall mortality in all studies including data on this outcome according to the presence of moderate/severe PVL vs. no/mild PVL as measured by angiography—we did not find data for the other outcomes or other scenarios such as mild PVL vs. no/trivial PVL. The data of 1567 patients (no/mild PVL: 1358 patients; moderate/severe PVL: 209 patients) from 5 studies were pooled. Patients with moderate/severe PVL had a significantly higher risk of overall mortality (HR, 3.02; 95% CI, 2.3-3.97; *p* < 0.001).

### Analysis of Outcomes According to the Presence of PVL (Measured by Echocardiography) and Type of THV–Self-Expandable (SEV) vs. Balloon-Expandable Valves (BEV)

**Figure 8:**
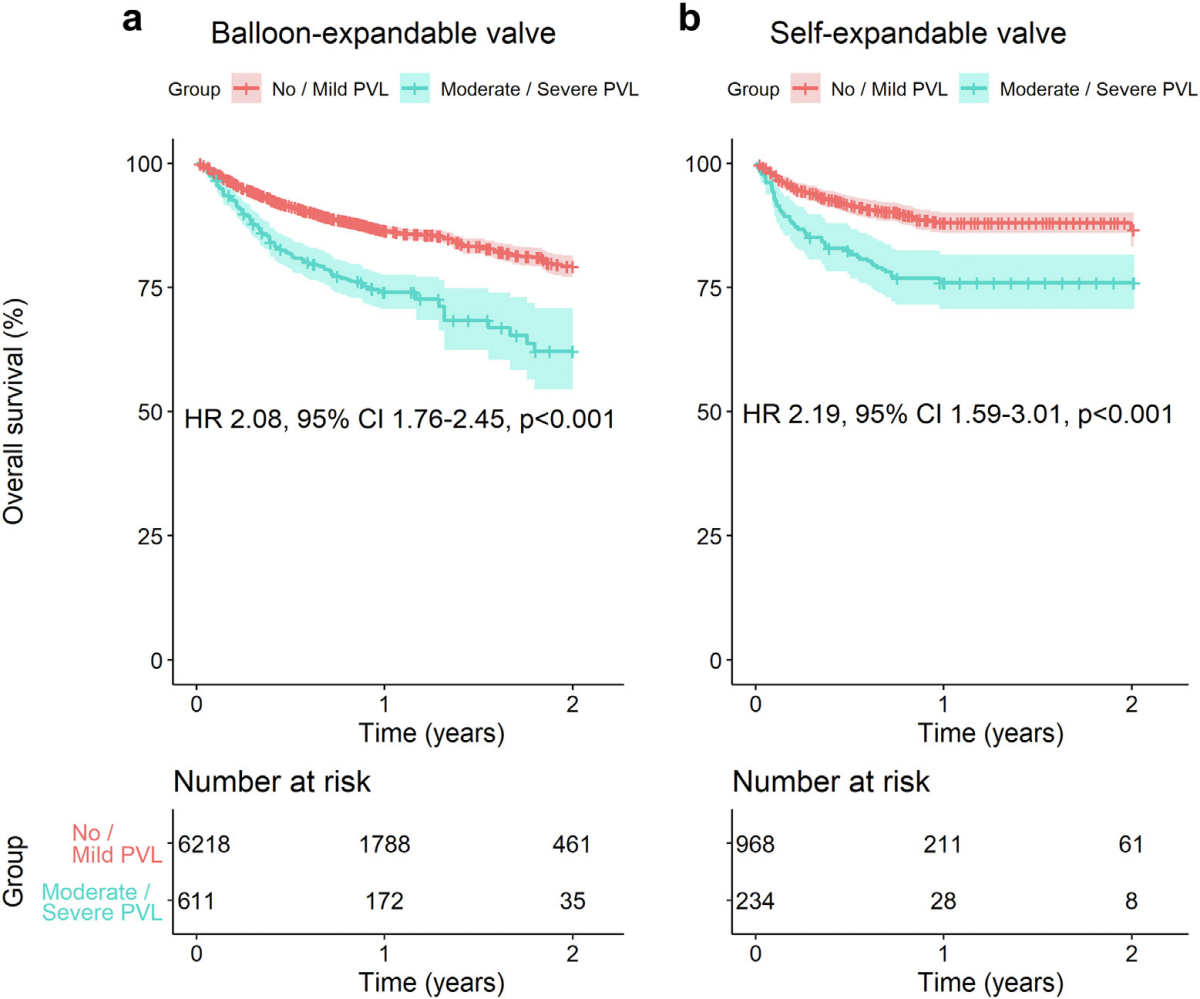
Pooled Kaplan-Meier curves showing the cumulative risk of all-cause mortality with balloon-expandable (a) and self-expandable (b) transcatheter heart valves for patients with moderate/severe PVL measured by echocardiography. LEGEND: Pooled Kaplan-Meier curves accompanied by their respective time-varying HRs with 95% CI at every given time during follow-up. Abbreviations: CI, confidence interval; HR, hazard ratio; PVL, paravalvular leak.

Figure 8 depicts the pooled Kaplan-Meier curve for the cumulative risk of overall mortality stratified by type of THV in all studies including data on this outcome according to the presence of moderate/severe PVL vs. no/mild PVL as measured by echocardiography. In these analyses, studies including either 100% SEV or 100% BEV were included. Patients with moderate/severe PVL had a significantly higher risk of overall mortality with both BEV (HR, 2.08; 95% CI, 1.76-2.45; *p* < 0.001) and SEV (HR, 2.19; 95% CI, 1.59-3.01; *p* < 0.001).

## Discussion

### Summary of Evidence

**Figure 9:**
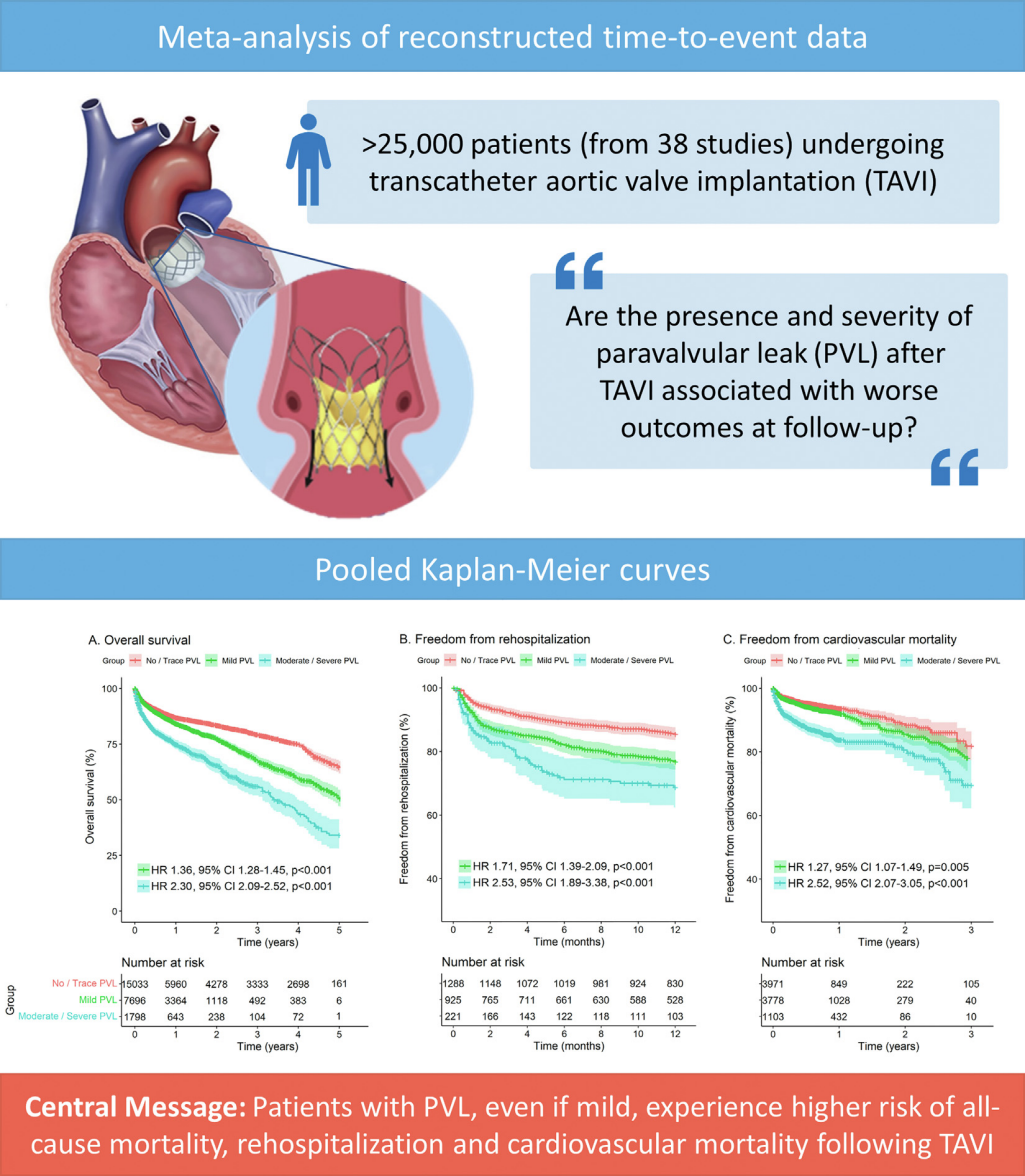
Graphical abstract - Both moderate/severe PVL and mild PVL were associated with increased risk of overall mortality, rehospitalization, and cardiovascular mortality during follow-up after TAVI. Abbreviations: CI, confidence interval; HR, hazard ratio; PVL, paravalvular leak.

To the best of our knowledge, this is the largest and first meta-analysis of reconstructed time-to-event data evaluating the impact of PVL on late outcomes of TAVI. (Figure 9). We found that not only moderate/severe PVL, but also mild PVL, is associated with increased risk of adverse events, including lower survival and higher risk of rehospitalization and cardiovascular mortality in patients after TAVI. As expected, the impact of moderate/severe PVL on outcomes is more important than that of mild PVL. However, the impact of mild PVL appears to increase with follow-up time. Furthermore, once moderate/severe PVL is present after TAVI, its negative impact on outcomes seems to be present in both BEV and SEV.

### Impact of PVL After TAVI

Moderate/severe PVL has been repeatedly associated with higher risk of death after TAVI.^15^, ^16^, ^17^, ^18^ The acute nature of postprocedural PVL in patients with preexisting severe aortic stenosis and thus whose left ventricle is not adapted to volume overload may explain the important detrimental impact of a moderate AR in these patients. Although chronic aortic regurgitation can remain asymptomatic for years even in the presence of left ventricular enlargement and/or dysfunction, acute aortic regurgitation secondary to PVL in the context of a left ventricle not “preconditioned” to volume overload can rapidly lead to decompensated heart failure and adverse events.

As a matter of fact, the peak of the HR for rehospitalization occurred early in the postprocedural period PVL (Figure 3b), when the mechanisms of LV compensation to volume overload are not yet established. The HR for rehospitalization stabilizes thereafter and converges toward the neutral value of 1.0, potentially due to the development of LV compensatory mechanisms. The impact of PVL on mortality nonetheless persists and even increases over time.

Owing to the extension of indications of TAVI in patients at lower risk and younger patients with long life expectancy,^19^ the potential negative impact of mild PVL on long-term outcomes may have important implications regarding the lifelong management of patients with aortic valve stenosis.^20^

Trials in high-risk surgical patients generally reported a negative impact of mild PVL on 5-year survival.^21^^,^^22^ A post hoc analysis of the PARTNER-2 trial, which included patients at intermediate surgical risk, failed to demonstrate the association of mild PVL with increased risk of 5-year mortality.^23^ Nevertheless, a slight but constant separation of the Kaplan-Meier curves for all-cause mortality between patients with mild PVL vs. those with none/trace PVL could be observed beyond 2 years of follow-up, suggesting a possibly latent negative effect of mild PVL on long-term survival.^23^ Our study, which has a much larger sample size with longer follow-up, was able to demonstrate the presence of a negative effect of mild PVL on outcomes.

### Clinical Implications

Our results have important implications for the management of patients with an indication of aortic valve replacement and suggest that a particular effort should be made to avoid any degree of PVL, even mild, in patients undergoing TAVI, and especially patients with longer life expectancy. SAVR might probably be preferred over TAVI in patients at high risk for PVL such as patients with severe calcification extending in the LVOT or patients with a bicuspid aortic valve having risk features for PVL such as calcified raphe and/or extensive valve calcification.^5^, ^6^, ^7^, ^8^^,^^24^ Montalto et al.^4^ showed that TAVI is a feasible option among selected patients with bicuspid anatomy, but the higher rates of moderate/severe PVL observed in the bicuspid population (in comparison with patients with trileaflet aortic valve) warrant caution and further evidence. Although there is evidence showing lower risk of PVL with new generation BEVs when compared with new generation SEVs,^7^ our results indicate that the presence of moderate/severe PVL after TAVI is detrimental with both BEVs and SEVs. Furthermore, in light of our results, re-intervention (balloon post-dilation, valve-in-valve, or closure with a plug) should be considered in patients who have undergone TAVI and present with moderate/severe PVL or mild PVL and evidence of heart failure (as manifested by rehospitalizations).

### Study Limitations

Firstly, considering the limitations of the approach used in this study, our meta-analysis of Kaplan-Meier-derived IPD has rather a hypothesis-generating nature. The method used does not enable us to establish a causal nexus between the presence of PVL and clinical events; however, it reveals an association with some biological plausibility.

Secondly, most of the studies comprised unadjusted cohorts, which are more prone to biases; however, the pooled effect size of subgroup analyses for mild and moderate/severe PVL (as opposed to any PVL) showed consistent results: that is, negative impact of PVL on outcomes regardless of severity. Because some studies did not report the difference in baseline characteristics between groups, there is a possibility that some patients had more comorbidities compared to the other arm and, therefore, had worse survival. The timing when PVL was measured may have influenced the outcomes. Although we did our best to eliminate overlapping data, some unmeasured overlap may remain in the multicentric studies coming from various countries. Since only one study classified PVL with cardiac magnetic resonance imaging, we do not know what the result would have been if we had more studies measuring PVL with this potentially more accurate method. Since this is a Kaplan-Meier-derived IPD meta-analysis and we do not have access to the source datasets which were used to generate the original publications, we were unable to analyze the impact of possible modulating factors on our results, such as age, sex, presence of comorbidities, left ventricle function, LVOT calcification, presence of bicuspid aortic valve, etc.

Another aspect to be highlighted is the low number of patients at risk following 2 to 3 years. The long-term effect of PVL is not adequately powered and, therefore, the study results are limited to short-term outcomes. Furthermore, THVs from different generations were mixed in most of the original samples and this might also impact outcomes in terms of survival.

## Conclusions

Patients with any degree of PVL experience higher risk of all-cause mortality, rehospitalization, and cardiovascular mortality after TAVI. Not only moderate/severe but also mild PVL is associated with increased risk of adverse events, and the impact of mild PVL appears to increase with time. These findings provide support to the implementation of procedural strategies to avoid any degree of PVL at the time of TAVI and for the consideration of re-intervention in patients with moderate/severe, and potentially even mild PVL if associated with heart failure, after TAVI.

## Ethics Statement

The research reported has adhered to the relevant ethical guidelines.

## Funding

Sharpe - Strumia Reuter Foundation (Bryn Mawr Hospital). Michel Pompeu Sá receives support from 10.13039/100010261The Thoracic Surgery Foundation (charitable arm of The Society of Thoracic Surgeons–STS) through the TSF Every Heartbeat Matters Global Structural Heart Fellowship Award for the project “Structural Heart/Minimally Invasive Cardiac Surgery”.

## Disclosure Statement

Philippe Pibarot has echocardiography Core Laboratory contracts with Edwards Lifesciences, for which he receives no direct compensation. Marie-Annick Clavel has a computed tomography Core Laboratory contract with Edwards Lifesciences, for which she receives no direct compensation, and has received a research grant from Medtronic. Basel Ramlawi has received financial support from Medtronic, Corcym, and AtriCure. The other authors had no conflicts to declare.

## Supplementary Material

Supplemental Table 1

Supplemental Reference List
