# Supplementary material for: Impact of Paravalvular Leak on Outcomes After Transcatheter Aortic Valve Implantation: Meta-Analysis of Kaplan-Meier-derived Individual Patient Data
Source: Struct Heart. 2022 Nov 14;7(2):100118. doi: 10.1016/j.shj.2022.100118 (PMC10236885; doi:10.1016/j.shj.2022.100118)
Supplement: Supplemental Table 1 [file mmc1.docx]

**Supplemental Table 1 – Characteristics of populations and studies included**

| **Study** | Design | Age (mean) | Female sex (%) | Transfemoral access  (%) | Surgical risk score (mean) | BEV (%) | SEV  (%) | MEV  (%) |
| --- | --- | --- | --- | --- | --- | --- | --- | --- |
| **Abdel-Wahab 2014** | R-M | 81.5 | 61 | 98 | 21* | 30 | 70 | NR |
| **Abdelgani 2017** | R-M | 81.4 | 25.5 | 94 | 12.7 | 31 | 67 | 2 |
| **Buzzatti 2017** | R-S | 80.6 | 55 | 87 | 5.6 | 55 | 35 | 6 |
| **Chieffo 2013** | R-M | 81.2 | 52.8 | 100 | 8.5 | 42.9 | 57.1 | 0 |
| **Collas 2017** | P-S | 83 | 54 | 84 | 5.3 | 0 | 100 | 0 |
| **D’Onofrio 2016** | R-S | 80 | 46 | 54 | 17.3* | 75 | 25 | 0 |
| **De Brito 2014** | P-M | 82 | 62 | 96 | 14 | 14 | 86 | 0 |
| **De Carlo 2013** | R-M | NR | NR | NR | NR | 0 | 100 | 0 |
| **Duncan 2015** | R-M | 82 | 46 | 68.4 | 18.5* | 47.5 | 52.5 | 0 |
| **Dworakowski 2014** | R-M | 81 | 47 | 67.7 | 21.6* | 52.7 | 47.2 | 0 |
| **Escarcega 2015** | R-S | 83 | 55.7 | NR | 10.1 | NR | NR | 0 |
| **Hayashida 2012** | R-S | 83.4 | 61.5 | NR | 7.9 | 86.8 | 13.2 | 0 |
| **Herrmann 2016** | R-M | 82.7 | 42 | 84 | 8.4 | 100 | 0 | 0 |
| **Ielasi 2015** | R-S | 79.7 | 49 | 81 | 24* | 60.2 | 39.8 | 0 |
| **Jelez-Valero 2014** | R-M | 81 | 52 | 73 | 7.7 | 56 | 43 | 0 |
| **Jilaihawi**  **2015** | R-S | 86 | 48.3 | 88.4 | 10.3 | 100 | 0 | 0 |
| **Jones 2016** | R-S | 80.6 | 42 | 100 | 9.7 | 100 | 0 | 0 |
| **Kodali 2015** | R-M | 84 | 48 | 58 | 11.2 | 100 | 0 | 0 |
| **Laakso 2020** | R-M | 81.2 | 55 | 91 | 5 | NR | NR | NR |
| **Lemos et al, 2012** | R-M | 82.5 | NR | 33 | 10 | 0 | 100 | 0 |
| **Makkar 2020** | P-M | 81.6 | 45.8 | 76.3 | 5.1 | 100 | 0 | 0 |
| **Meneguz-Moreno 2017** | R-M | 82.1 | 55 | 89.2 | 6.6 | 38.3 | 61.7 | 0 |
| **Miyazaki 2015** | R-S | 79.5 | 51 | 85.6 | 18* | 56 | 37.5 | 6.6 |
| **Mohr 2014** | R-M | 80.9 | 56 | 69.5 | NR | NR | NR | NR |
| **Okuno 2022** | R-S | 82.3 | 52.1 | 85.4 | 6 | 49.4 | 44.8 | 5.7% |
| **Patsalis 2013** | R-S | 80.7 | 58 | 94.6 | 7.5 | 47.3 | 52.7 | 0 |
| **Pibarot 2017** | R-M | 81.6 | 39.7 | 86.9 | 6.4 | 100 | 0 | 0 |
| **Ribeiro 2016** | P-M | 79.7 | 49 | 69.6 | 5.2 | 95.6 | 4.4 | 0 |
| **Schoechlin 2017** | P-M | 82 | 55.5 | 99 | 21.9* | 79.1 | 20.9 | 0 |
| **Sinning 2012** | R-S | 80.5 | 52 | 91.8 | 9.8 | 0 | 100 | 0 |
| **Søndergaard 2016** | P-M | 79.1 | 47 | 96 | 3.0 | 0 | 100 | 0 |
| **Thourani 2016** | P-M | 82 | 38 | 88 | 5.2 | 100 | 0 | 0 |
| **Toggweiler 2013** | R-S | 83 | 47 | 73 | 9 | 100 | 0 | 0 |
| **Unbehaun 2014** | R-S | 79 | 53 | 0 (100% trans-apical) | 8 | 100 | 0 | 0 |
| **Van Belle 2014** | P-M | 83 | 49 | 75.3 | 21.5* | 67.6 | 32.4 | 0 |
| **Yared 2012** | P-M | NR | NR | 58.3 | NR | 100 | 0 | 0 |
| **Yoshijima 2020** | R-M | 85 | 73 | 79.7 | 6.6 | 91.5 | 8.4 | 0 |

LEGEND: O – observational; P – prospective; R – retrospective; M – multicentric; S – single-center study; NR – non-reported; BEV – balloon-expandable valve; SEV – self-expandable valve; MEV – mechanically expandable valve; PVL – paravalvular leakage; *STS n/a, only Logistic Euro Score
