# Supplementary material for: Impact of Paravalvular Leak on Outcomes After Transcatheter Aortic Valve Implantation: Meta-Analysis of Kaplan-Meier-derived Individual Patient Data
Source: Struct Heart. 2022 Nov 14;7(2):100118. doi: 10.1016/j.shj.2022.100118 (PMC10236885; doi:10.1016/j.shj.2022.100118)
Supplement: Supplemental Reference List [file mmc2.docx]

LIST OF STUDIES

1. Okuno T, Tomii D, Heg D, Lanz J, Praz F, Stortecky S, Reineke D, Windecker S, Pilgrim T. Five-year outcomes of mild paravalvular regurgitation after transcatheter aortic valve implantation. EuroIntervention. 2021. doi: 10.4244/EIJ-D-21-00784. Online ahead of print.

2. Makkar RR, Thourani VH, Mack MJ, Kodali SK, Kapadia S, Webb JG, Yoon SH, Trento A, Svensson LG, Herrmann HC, Szeto WY, Miller DC, Satler L, Cohen DJ, Dewey TM, Babaliaros V, Williams MR, Kereiakes DJ, Zajarias A, Greason KL, Whisenant BK, Hodson RW, Brown DL, Fearon WF, Russo MJ, Pibarot P, Hahn RT, Jaber WA, Rogers E, Xu K, Wheeler J, Alu MC, Smith CR, Leon MB; PARTNER 2 Investigators. Five-Year Outcomes of Transcatheter or Surgical Aortic-Valve Replacement. N Engl J Med. 2020;382:799-809.

3. Yoshijima N, Yanagisawa R, Hase H, et al. Update on the clinical impact of mild aortic regurgitation after transcatheter aortic valve implantation: Insights from the Japanese multicenter OCEAN-TAVI registry. Catheter Cardiovasc Interv. 2020;95:35–44.

4. Laakso T, Laine M, Moriyama N, Dahlbacka S, Airaksinen J, Virtanen M et al. Impact of paravalvular regurgitation on the mid-term outcome after transcatheter and surgical aortic valve replacement. Eur J Cardiothorac Surg 2020;58:1145–52.

5. Schoechlin S, Brennemann T, Allali A, et al. Hemodynamic classification of paravalvular leakage after transcatheter aortic valve implantation compared with angiographic or echocardiographic classification for prediction of 1-year mortality. Catheter Cardiovasc Interv. 2018;93:E56–6E3.

6. Gleason TG, Reardon MJ, Popma JJ, Deeb GM, Yakubov SJ, Lee JS, Kleiman NS, Chetcuti S, Hermiller JB Jr, Heiser J, Merhi W, Zorn GL 3rd, Tadros P, Robinson N, Petrossian G, Hughes GC, Harrison JK, Conte JV, Mumtaz M, Oh JK, Huang J, Adams DH; CoreValve U. S. Pivotal High Risk Trial Clinical Investigators. 5-Year Outcomes of Self Expanding Transcatheter Versus Surgical Aortic Valve Replacement in High-Risk Patients. J Am Coll Cardiol. 2018;72:2687-96.

7. Pibarot P, Hahn RT, Weissman NJ, Arsenault M, Beaudoin J, Bernier M, Dahou A, Khalique OK, Asch FM, Toubal O, Leipsic J, Blanke P, Zhang F, Parvataneni R, Alu M, Herrmann H, Makkar R, Mack M, Smalling R, Leon M, Thourani VH, Kodali S. Association of Paravalvular Regurgitation With 1-Year Outcomes After Transcatheter Aortic Valve Replacement With the SAPIEN 3 Valve. JAMA Cardiol. 2017;2(11):1208-1216.

8. Meneguz-Moreno RA, Castro-Filho A, Ramos AIO, Zumarraga M, Bihan DL, Barretto R, Siqueira DAA, Abizaid AAC, Sousa AGMR, Sousa JE. Progression and Prognosis of Paravalvular Regurgitation After Transcatheter Aortic Valve Implantation. Arq Bras Cardiol. 2017; 109(6):590-598)

9. Abdelghani M, Tateishi H, Miyazaki Y, Cavalcante R, Soliman OII, Tijssen JG, de Winter RJ, Baan J Jr, Onuma Y, Campos CM, Leite RS, Mangione JA, Abizaid A, Lemos PA, de Brito FS Jr, Serruys PW. Angiographic assessment of aortic regurgitation by video-densitometry in the setting of TAVI: Echocardiographic and clinical correlates. Catheter Cardiovasc Interv. 2017 Oct 1;90(4):650-659.

10. Buzzatti N, Castiglioni A, Agricola E, Barletta M, Stella S, Giannini F, Regazzoli D, Mangieri A, Ancona M, Spagnolo P, Chieffo A, Montorfano M, Alfieri O, Colombo A, Latib A. Five-year evolution of mild aortic regurgitation following transcatheter aortic valve implantation: early insights from a single-centre experience. Interact Cardiovasc Thorac Surg. 2017 Jul 1;25(1):75-82.

11. Collas VM, Paelinck BP, Rodrigus IE, Vrints CJ, Bosmans JM. Aortic regurgitation after transcatheter aortic valve implantation (TAVI) - Angiographic, echocardiographic and hemodynamic assessment in relation to one year outcome. Int J Cardiol. 2015;194:13-20.

12. D’Onofrio A, Facchin M, Besola L, Manzan E, Tessari C, Bizzotto E, Bianco R, Tarantini G, Napodano M, Fraccaro C, Buja P, Covolo E, Yzeiraj E, Pittarello D, Isabella G, Iliceto S, Gerosa G. Intermediate clinical and hemodynamic outcomes after transcatheter aortic valve implantation. Ann Thorac Surg 2016;101:881–888.

13. Jones BM, Tuzcu EM, Krishnaswamy A, Popovic Z, Mick S, Roselli EE, Gul S, Devgun J, Mistry S, Jaber WA, Svensson LG, Kapadia SR. Prognostic significance of mild aortic regurgitation in predicting mortality after transcatheter aortic valve replacement. J Thorac Cardiovasc Surg 2016;152:783–790.

14. Ribeiro HB, Orwat S, Hayek SS, Larose E, Babaliaros V, Dahou A, Le Ven F, Pasian S, Puri R, Abdul-Jawad Altisent O, Campelo-Parada F, Clavel MA, Pibarot P, Lerakis S, Baumgartner H, Rodes-Cabau J. Cardiovascular magnetic resonance to evaluate aortic regurgitation after transcatheter aortic valve replacement. J Am Coll Cardiol 2016;68:577–585.

15. Søndergaard L, Steinbr€uchel DA, Ihlemann N, Nissen H, Kjeldsen BJ, Petursson P, Ngo AT, Olsen NT, Chang Y, Franzen OW, Engstrøm T, Clemmensen P, Olsen PS, Thyregod HG. Two year outcomes in patients with severe aortic valve stenosis randomized to transcatheter versus surgical aortic valve replacement: The allcomers nordic aortic valve intervention randomized clinical trial. Circ Cardiovasc Interv 2016;9:e003665.

16. Thourani VH, Kodali S, Makkar RR, Herrmann HC, Williams M, Babaliaros V, Smalling R, Lim S, Malaisrie SC, Kapadia S, Szeto WY, Greason KL, Kereiakes D, Ailawadi G, Whisenant BK, Devireddy C, Leipsic J, Hahn RT, Pibarot P, Weissman NJ, Jaber WA, Cohen DJ, Suri R, Tuzcu EM, Svensson LG, Webb JG, Moses JW, Mack MJ, Miller DC, Smith CR, Alu MC, Parvataneni R, D’Agostino RB, Jr., Leon MB. Transcatheter aortic valve replacement versus surgical valve replacement in intermediate-risk patients: A propensity score analysis. Lancet 2016;387:2218–2225.

17. Herrmann HC, Thourani VH, Kodali SK, Makkar RR, Szeto WY,Anwaruddin S, Desai N, Lim S, Malaisrie SC, Kereiakes DJ, Ramee S, Greason KL, Kapadia S, Babaliaros V, Hahn RT, Pibarot P, Weissman NJ, Leipsic J, Whisenant BK, Webb JG, Mack MJ, Leon MB; PARTNER Investigators. One-year clinical outcomes with SAPIEN 3 transcatheter aortic valve replacement in high-risk and inoperable patients with severe aortic stenosis. Circulation 2016;134:130–140.

18. Ielasi A, Latib A, Sacco FM, Costopoulos C, Figini F, Grimaldi A, Naim C, Maisano F, Chieffo A, Montorfano M, Alfieri O, Colombo A. Impact and natural history of postprocedural aortic regurgitation on early and midterm mortality following transcatheter aortic valve implantation in high-risk patients with severe aortic stenosis. J Cardiovasc Med (Hagerstown) 2015;16:286–295.

19. Duncan A, Ludman P, Banya W, Cunningham D, Marlee D, Davies S, Mullen M, Kovac J, Spyt T, Moat N. Long-term outcomes after transcatheter aortic valve replacement in high-risk patients with severe aortic stenosis: the U.K. Transcatheter Aortic Valve Implantation Registry. JACC Cardiovasc Interv. 2015;8(5):645-53.

20. Escarcega RO, Lipinski MJ, Baker NC, Magalhaes MA, Minha S, Torguson R, Chen F, Ben Dor I, Satler LF, Pichard AD, Corso P, Waksman R. Analysis of long-term survival following transcatheter aortic valve implantation from a single high-volume center. Am J Cardiol 2015;116:256–263.

21. Miyazaki S, Agricola E, Panoulas VF, Slavich M, Giustino G, Miyazaki T, Figini F, Latib A, Chieffo A, Montorfano M, Margonato A, Maisano F, Alfieri O, Colombo A. Influence of baseline ejection fraction on the prognostic value of paravalvular leak after transcatheter aortic valve implantation. Int J Cardiol 2015;190:277–281.

22. Jilaihawi H, Chakravarty T, Shiota T, Rafique A, Harada K, Shibayama K, Doctor N, Kashif M, Nakamura M, Mirocha J, Rami T, Okuyama K, Cheng W, Sadruddin O, Siegel R, Makkar RR. Heartrate adjustment of transcatheter haemodynamics improves the prognostic evaluation of paravalvular regurgitation after transcatheter aortic valve implantation. EuroIntervention 2015;11:456–464.

23. Kodali S, Pibarot P, Douglas PS, Williams M, Xu K, Thourani V, Rihal CS, Zajarias A, Doshi D, Davidson M, Tuzcu EM, Stewart W, Weissman NJ, Svensson L, Greason K, Maniar H, Mack M, Anwaruddin S, Leon MB, Hahn RT. Paravalvular regurgitation after transcatheter aortic valve replacement with the Edwards Sapien valve in the PARTNER trial: Characterizing patients and impact on outcomes. Eur Heart J 2015;36:449–456.

24. Abdel-Wahab M, Neumann FJ, Mehilli J, Frerker C, Richardt D, Landt M, Jose J, Toelg R, Kuck KH, Massberg S, Robinson DR, El- Mawardy M, Richardt G; CHOICE Investigators. 1 Year outcomes after transcatheter aortic valve replacement with balloonexpandable versus self expandable valves: results from the CHOICE randomized clinical trial. J Am Coll Cardiol 2015;66:791–800.

25. de Brito FS Jr, Carvalho LA, Sarmento-Leite R, Mangione JA, Lemos P, Siciliano A, Caramori P, São Thiago L, Grube E, Abizaid A; Brazilian TAVI Registry investigators. Outcomes and predictors of mortality after transcatheter aortic valve implantation: results of the Brazilian registry. Catheter Cardiovasc Interv 2015;85(5):E153-62.

26. Dworakowski R, Wendler O, Halliday B, Ludman P, DeBelder M, Ray S, Moat N, Kovac J, Spyt T, Trivedi U, Hildick-Smith D, Blackman D, Marlee D, Cunningham D, MacCarthy PA. Device-dependent association between paravalvar aortic regurgitation and outcome after TAVI. Heart 2014;100:1939–1945.

27. Jerez-Valero M, Urena M, Webb JG, Tamburino C, Munoz-Garcia AJ, Cheema A, Dager AE, Serra V, Amat-Santos IJ, Barbanti M, Immè S, Alonso Briales JH, Al Lawati H, Benitez LM, Cucalon AM, Garcia del Blanco B, Revilla A, Dumont E, Barbosa Ribeiro H, Nombela-Franco L, Bergeron S, Pibarot P, Rod es-Cabau J. Clinical impact of aortic regurgitation after transcatheter aortic valve replacement: insights into the degree and acuteness of presentation. JACC Cardiovasc Interv 2014;7:1022–1032.

28. Mohr FW, Holzhey D, Möllmann H, Beckmann A, Veit C, Figulla HR, Cremer J, Kuck KH, Lange R, Zahn R, Sack S, Schuler G, Walther T, Beyersdorf F, B€ohm M, Heusch G, Funkat AK, Meinertz T, Neumann T, Papoutsis K, Schneider S, Welz A, Hamm CW; GARY Executive Board. The German Aortic Valve Registry: 1-Year results from 13,680 patients with aortic valve disease. Eur J Cardiothorac Surg 2014;46:808–816.

29. Unbehaun A, Pasic M, Kukucka M, Mladenow A, Solowjowa N, Dreysse S, Drews T, Penkalla A, Hetzer R, Buz S. Transapical aortic valve implantation: predictors of leakage and impact on survival: An update. Ann Thorac Surg 2014;98:1308–1315.

30. Van Belle E, Juthier F, Susen S, Vincentelli A, Iung B, Dallongeville J, Eltchaninoff H, Laskar M, Leprince P, Lievre M, Banfi C, Auffray JL, Delhaye C, Donzeau-Gouge P, Chevreul K, Fajadet J, Leguerrier A, Prat A, Gilard M, Teiger E; FRANCE 2 Investigators. Postprocedural aortic regurgitation in balloon-expandable and self-expandable transcatheter aortic valve replacement procedures: Analysis of predictors and impact on long-term mortality: insights from the FRANCE2 Registry. Circulation 2014;129:1415–1427.

31. Chieffo A, Buchanan GL, Van Mieghem NM, Tchetche D, Dumonteil N, Latib A, van der Boon RM, Vahdat O, Marcheix B, Farah B, Serruys PW, Fajadet J, Carri e D, de Jaegere PP, Colombo A. Transcatheter aortic valve implantation with the Edwards SAPIEN versus the Medtronic CoreValve Revalving system devices: A multicenter collaborative study: the PRAGMATIC Plus Initiative (Pooled-RotterdAm- Milano-Toulouse In Collaboration). J Am Coll Cardiol 2013;61: 830–836.

32. Toggweiler S, Humphries KH, Lee M, Binder RK, Moss RR, Freeman M, et al., 5-year outcome after transcatheter aortic valve implantation, J. Am. Coll. Cardiol. 2013;61:413–419.

33. Patsalis PC, Konorza TF, Al-Rashid F, Plicht B, Riebisch M, Wendt D, Thielmann M, Jakob H, Eggebrecht H, Heusch G, Erbel R, Kahlert P. Incidence, outcome and correlates of residual paravalvular aortic regurgitation after transcatheter aortic valve implantation and importance of haemodynamic assessment. EuroIntervention 2013;8:1398–1406.

34. De Carlo M, Giannini C, Fiorina C, Bedogni F, Napodano M, Klugmann S, Tamburino C, Maisano F, Santoro G, Ettori F, Petronio AS. Paravalvular leak after CoreValve implantation in the Italian Registry: predictors and impact on clinical outcome. Int J Cardiol. 2013 12;168(5):5088-9.

35. Hayashida K, Lefèvre T, Chevalier B, Hovasse T, Romano M, Garot P, Bouvier E, Farge A, Donzeau-Gouge P, Cormier B, Morice MC. Impact of post-procedural aortic regurgitation on mortality after transcatheter aortic valve implantation. JACC Cardiovasc Interv 2012;5:1247–1256.

36. Lemos PA, Saia F, Mariani J, Jr., Marrozzini C, Filho AE, Kajita LJ, Ciuca C, Taglieri N, Bordoni B, Moretti C, Palmerini T, Dracoulakis MD, Jatene FB, Kalil-Filho R, Marzocchi A. Residual aortic regurgitation is a major determinant of late mortality after transcatheter aortic valve implantation. Int J Cardiol 2012;157:288–289.

37. Sinning JM, Stundl A, Pingel S, Weber M, Sedaghat A, Hammerstingl C, Vasa-Nicotera M, Mellert F, Schiller W, Kovac J, Welz A, Grube E, Werner N, Nickenig G. Pre-procedural hemodynamic status improves the discriminatory value of the aortic regurgitation index in patients undergoing transcatheter aortic valve replacement. JACC Cardiovasc Interv 2016;9:700–711.

38. Yared K, Garcia-Camarero T, Fernandez-Friera L, Llano M, Durst R, Reddy AA, O’Neill WW, Picard MH. Impact of aortic regurgitation after transcatheter aortic valve implantation: results from the REVIVAL trial. JACC Cardiovasc Imaging 2012;5:469–477.
